# Timing matters: the processing of pitch relations

**DOI:** 10.3389/fnhum.2014.00387

**Published:** 2014-06-11

**Authors:** Annekathrin Weise, Sabine Grimm, Nelson J. Trujillo-Barreto, Erich Schröger

**Affiliations:** ^1^Kognitive einschließlich Biologische Psychologie, Institut für Psychologie, Universität LeipzigLeipzig, Germany; ^2^Institute for Brain, Cognition and Behaviour (IR3C), University of BarcelonaBarcelona, Spain; ^3^Cognitive Neuroscience Research Group, Department of Psychiatry and Clinical Psychobiology, University of BarcelonaBarcelona, Spain; ^4^Neuroinformatics Department, Cuban Neuroscience CentreCiudad Habana, Cuba

**Keywords:** abstract regularities, automatic processing, frontal generators, mismatch negativity, supratemporal generators, temporal window of integration

## Abstract

The human central auditory system can automatically extract abstract regularities from a variant auditory input. To this end, temporarily separated events need to be related. This study tested whether the timing between events, falling either within or outside the temporal window of integration (~350 ms), impacts the extraction of abstract feature relations. We utilized tone pairs for which tones within but not across pairs revealed a constant pitch relation (e.g., pitch of second tone of a pair higher than pitch of first tone, while absolute pitch values varied across pairs). We measured the mismatch negativity (MMN; the brain’s error signal to auditory regularity violations) to second tones that rarely violated the pitch relation (e.g., pitch of second tone lower). A *Short* condition in which tone duration (90 ms) and stimulus onset asynchrony between the tones of a pair were short (110 ms) was compared to two conditions, where this onset asynchrony was long (510 ms). In the *Long Gap* condition, the tone durations were identical to *Short* (90 ms), but the silent interval was prolonged by 400 ms. In *Long Tone*, the duration of the first tone was prolonged by 400 ms, while the silent interval was comparable to *Short* (20 ms). Results show a frontocentral MMN of comparable amplitude in all conditions. Thus, abstract pitch relations can be extracted even when the within-pair timing exceeds the integration period. Source analyses indicate MMN generators in the supratemporal cortex. Interestingly, they were located more anterior in *Long Gap* than in *Short* and *Long Tone*. Moreover, frontal generator activity was found for *Long Gap* and *Long Tone*. Thus, the way in which the system automatically registers irregular abstract pitch relations depends on the timing of the events to be linked. Pending that the current MMN data mirror established abstract rule representations coding the regular pitch relation, neural processes building these templates vary with timing.

## INTRODUCTION

An important skill of the central auditory system is to extract regularities from the ever-changing acoustic environment. Such regularities can be rather abstract in nature, for instance, when the features of successive sound events possess an invariant relationship while absolute features vary from one event to the next. The ability to extract abstract regularities has been evidenced by plenty of studies (Saarinen et al., 1992; Korzyukov et al., 2003; Paavilainen et al., 2003; Carral et al., 2005; van Zuijen et al., 2006; Schröger et al., 2007; for a recent review, see Paavilainen, 2013). Given the fact that temporal aspects play a crucial role in sequential auditory processing (Atienza et al., 2003; Shinozaki et al., 2003; Grimm and Schröger, 2005; Sussman and Gumenyuk, 2005), this study aimed at systematically testing the impact of timing on the extraction of abstract regularities.

A powerful tool to research automatic regularity extraction is the mismatch negativity (MMN; Näätänen et al., 1978; for reviews, see, e.g., Näätänen et al., 2007; Winkler and Czigler, 2012) component of the event-related potential (ERP). Typically, MMN is elicited by auditory events (deviants) that violate an auditory rule which was established on the basis of a regularity inherent to the preceding sounds (standards). MMN becomes visible in the ERP as a more negative deflection for the deviant than for the standard at a latency of about 100 to 200 ms following the deviance with maximal amplitudes over frontocentral sites. When data are referenced against an electrode placed at the nose, the negative frontocentral MMN is often accompanied by a positive mastoidal MMN (Picton et al., 2000; Schröger, 2005). The latter originates from the dipolar supratemporal generators in auditory areas, whereas the frontocentral MMN receives contributions from supratemporal generators as well as from frontal generators (Alho, 1995; Deouell, 2007).

Crucially for the present purpose, MMN is not only sensitive to violations of simple rules (e.g., standard tones with same pitch values; Näätänen et al., 1978), but even to violations of more complex rules. These rules may be built, for instance, on the basis of tonal patterns (Winkler and Schröger, 1995; Sussman and Gumenyuk, 2005), feature conjunctions (Ritter et al., 1995; Gomes et al., 1997), or abstract feature relations (Saarinen et al., 1992; Tervaniemi et al., 1994). By applying an abstract pitch regularity MMN approach numerous studies found, for instance, MMN to the violation of an invariant pitch relation between two tones forming a pair (e.g., standard pair: rising pitch, deviant pair: falling pitch) while across pairs pitch values varied (Saarinen et al., 1992; Paavilainen et al., 2003; Carral et al., 2005; Schröger et al., 2007). Especially the results from approaches utilizing such complex rules corroborate the view that MMN is the outcome of a sensory memory comparison-based deviance detection process. According to this notion, the deviance detection system operates on the basis of auditory sensory memory representation. Based on the invariances inherent to the incoming auditory events the auditory system builds regularity representations from which predictions (stored as templates) about forthcoming events can be derived. If the prediction is not met by the incoming event, MMN is elicited (Winkler and Czigler, 2012; Schröger et al., 2013).

By utilizing the MMN we aimed at testing the impact of timing on the extraction of invariant abstract pitch relations. Especially the temporal window of integration (Yabe et al., 2001; Shinozaki et al., 2003; for reviews, see Cowan, 1984; Näätänen et al., 2011) might play a crucial role in this context. This function of auditory sensory memory, being triggered by auditory transients (e.g., tone onset or pitch transition within tones; Näätänen and Winkler, 1999; Weise et al., 2010, 2012), allows binding and relating successive auditory event information (Yabe et al., 2001; Shinozaki et al., 2003; for reviews, see Cowan, 1984; Näätänen and Winkler, 1999), thus aiding the establishment of regularity representations.

Given the limited time span of the temporal window of integration, ranging up to 350 ms, this window has been supposed to be responsible for the temporal constraint in establishing regularity representations (simple regularities: Näätänen et al., 2004; Grimm and Schröger, 2005; Hoonhorst et al., 2012; more complex regularities: Atienza et al., 2003; Sussman and Gumenyuk, 2005). For instance, the finding that a deviating feature (e.g., a brief modulation in pitch) within one of the repeatedly presented tones of constant pitch elicited MMN only when it occurred before 350 ms following tone onset, but not thereafter was linked to the temporal window of integration (Näätänen et al., 2004; Grimm and Schröger, 2005; Timm et al., 2011; Hoonhorst et al., 2012). Accordingly, the initial parts of a tone, falling within the integration period, can be integrated into a unitary sound representation, thus, enabling previous events (e.g., tone onset) to be related to subsequent ones (e.g., the deviating feature).

Not only for the formation of simple regularities, but even for more complex ones, such as pattern-like templates spanning a larger temporal scale, an impact of the temporal window of integration has been reported. Atienza et al. (2003), for instance, presented trains of six tones which alternated in pitch (e.g., ABABAB). The onset-to-onset interval between adjacent tones within this train was 120 ms. Rarely, the last tone of the preceding and the first tone of the following train were identical (e.g., ABABAB-**B** ABABA; “**B**” marks the irregular repetition; “-” highlights the offset-to-onset interval between adjacent trains). In different conditions, the silent interval between adjacent trains varied. Crucially, a repeating tone elicited MMN only for intervals not exceeding 300 ms enabling adjacent tones of successive trains to be bound together. Additional evidence for the impact of timing on the formation of regularity representations comes from another approach utilizing a five-tone pattern (A-A-A-A-B) being repeatedly presented (Sussman and Gumenyuk, 2005). Adjacent tones were separated by a constant onset-to-onset interval. In different conditions, the onset-to-onset interval varied. When this interval was 400 ms and above, B elicited MMN while no MMN was found when the interval lasted 200 ms. From this pattern of results they concluded that the short 200-ms interval allowed the A-A-A-A-B sequence to be encoded as a regular five-tone-pattern. Thus, there was no deviant in the auditory stimulation causing a mismatch between the templates of predicted and actual incoming event. However, if the onset-to-onset interval was 400 ms and longer, each tone (A and B) was extracted as a single event, causing the elicitation of MMN to the B tone (Sussman and Gumenyuk, 2005).

Based on the impact of timing in forming regularity representations of simple and pattern-like events (Atienza et al., 2003; Näätänen et al., 2004; Sussman and Gumenyuk, 2005; Hoonhorst et al., 2012) we aimed at determining whether the temporal window of integration plays also a crucial role for the extraction of abstract pitch relations, relevant for building corresponding regularity representations. One attempt addressing this topic has already been made (Paavilainen et al., 2003) by utilizing an abstract pitch MMN approach (Saarinen et al., 1992; Korzyukov et al., 2003; Paavilainen et al., 2003; Carral et al., 2005; Schröger et al., 2007). In different conditions, timing was varied by manipulating the onset-to-onset within-pair interval (110, 160, 210, or 260 ms). As MMN was of comparable size across conditions, Paavilainen et al. (2003) concluded that the extraction of abstract pitch relations does not depend on the temporal window of integration. A putative caveat in that approach (Paavilainen et al., 2003) is that the longest onset-to-onset within-pair interval being used (260 ms) does not necessarily exceed the integration period. Even though estimated under some stimulus settings to approximately 170–250 ms (Yabe et al., 2001; Shinozaki et al., 2003), the temporal window of integration has been suggested in other cases to last up to 350 ms (Cowan, 1984; Grimm and Schröger, 2005; Grimm et al., 2006). Thus, the outcome of this former study (Paavilainen et al., 2003) does not provide a final answer to the current research question whether or not timing affects the extraction of abstract pitch relations.

On the other hand, there exists evidence from (at least) two studies that abstract pitch relations between adjacent tones (which were not presented in pairs) can in principle be automatically extracted when the onset-to-onset interval exceeded the temporal window of integration (Tervaniemi et al., 1994; Bendixen and Schröger, 2008). One of the studies did not find a polarity inversed version of the present frontocentral MMN. The other study (Tervaniemi et al., 1994) did not analyze (and did not show) data recorded at the mastoids. It may thus be suspected that mastoid MMN was absent as well. However, mastoidal MMN was present for the violation of abstract pitch relations when the onset-to-onset interval between adjacent tones fell within the integration period (Schröger et al., 2007). This pattern of results seems to suggest that depending on the timing different mechanisms may be involved in extracting pitch relations. Anyway, the onset-to-onset (and offset-to-onset) interval has – to our knowledge – not yet been systematically varied in order to test this hypothesis.

Another timing-related aspect being in the scope of the present study is the role of the offset-to-onset interval between successive tones. This issue received less attention to date. Whereas in previous approaches timing between adjacent tones was manipulated by varying both onset-to-onset- and offset-to-onset interval (Alain et al., 1994; Atienza et al., 2003; Paavilainen et al., 2003; Sussman and Gumenyuk, 2005), this variation allowed no conclusions concerning the relevance of the offset-to-onset interval. That is, the role of the duration of the silent interval during which no information relevant for the extraction of the critical features is available needs still to be determined. As has been suggested for other transients like sounds’ onset or pitch transitions within a tone (onset: Yabe et al., 1997; Grimm and Schröger, 2005; pitch transition: Weise et al., 2010, 2012), even sound offset might act as a trigger for integration. Accordingly, the extraction of within-pair pitch relations should be undiminished when the offset-to-onset interval falls within the integration window.

**FIGURE 1:**
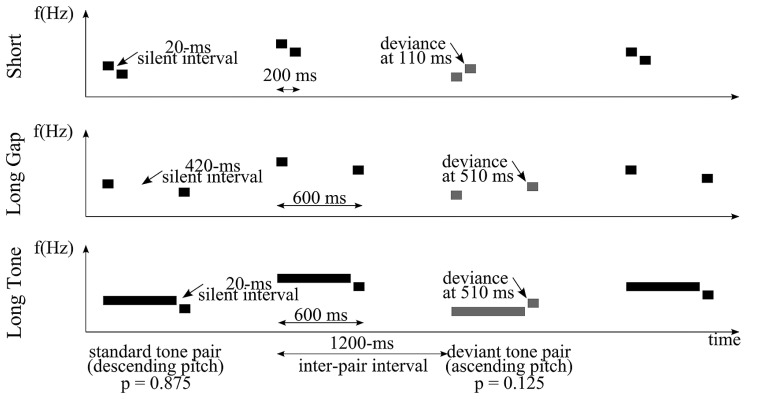
**Schematic representation of an exemplary stimulus sequence consisting of tone pairs for each condition: *Short* (top), *Long Gap* (middle), and *Long Tone* (bottom).** Here, the stimulation comprised an abstract, falling rule that was formed by a frequently presented standard tone pair (two black bars) featuring a constant descending pitch relation. A rare deviant pair (two gray bars) violates the rising rule in that it features a constant ascending pitch relation. To establish an abstract rule, tone pairs varied in their absolute pitch values from trial to trial. Inter-pair interval was 1200 ms. The silent within-pair interval and/or duration of the first tone of a pair varied across conditions.

To systematically test the impact of timing on the processing of abstract pitch relations we utilized a similar abstract pitch MMN approach as used by Paavilainen et al. (2003; see also Saarinen et al., 1992; Korzyukov et al., 2003; Carral et al., 2005; van Zuijen et al., 2006; Schröger et al., 2007). Crucially, we did not only vary the onset-to-onset within-pair interval but also the offset-to-onset within-pair interval, each being either within or beyond the range of integration window. To manipulate the onset-to-onset interval we varied the duration of the gap within the pair. To alter the offset-to-onset interval, we varied the duration of the first tone of the pair. This resulted in three conditions (*Short*, *Long Gap*, *Long Tone*; **Figure 1**), each of them utilizing pairs of tones presented with a constant inter-pair interval (1200 ms). In *Short*, pairs consisted of two 90-ms tones separated by a 20-ms gap. In *Long Gap* and *Long Tone*, the duration of the gap or the duration of the first tone, respectively, was considerably extended (by 400 ms). With respect to the conditions *Short* and *Long Gap* the variations led to identical durations of the first tone (90 ms) but diverging offset-to-onset within-pair intervals. Concerning *Short* and *Long Tone*, these variations resulted in identical (20 ms) offset-to-onset within-pair intervals but different durations of the first tones.

Based on previous findings reporting a temporal constraint in the formation of regularity representation (Atienza et al., 2003; Näätänen et al., 2004; Grimm and Schröger, 2005; Sussman and Gumenyuk, 2005; Hoonhorst et al., 2012), we expect a comparable bottleneck for the processing of abstract pitch relations. The critical temporal boundary is supposed to be defined by the temporal window of integration lasting up to 350 ms. Within this window, as it is the case in *Short* condition, we expect the deviance detection system to relate the two pitches easily, reflected in a distinct MMN amplitude for irregular pitch relations (Saarinen et al., 1992; Paavilainen et al., 2003; Schröger et al., 2007). The critical conditions are those in which the onset-to-onset within-pair interval or the offset-to-onset within-pair interval exceeds this window. In *Long Gap* condition, in which the onset-to-onset interval as well as the offset-to onset interval exceeds the integration period we expect the deviance detection system to have difficulties relating the pitches. This should be reflected in an attenuated MMN amplitude in *Long Gap* compared to *Short*. In *Long Tone*, in which the onset-to-onset interval but not the offset-to-onset interval exceeds the integration window, we derive two hypotheses: if the onset-to-onset interval matters (i.e., meaning that it is outside the temporal window of integration) for the extraction of abstract pitch relations, we expect a reduced MMN amplitude in *Long Tone* compared to *Short*. Alternatively, if the offset-to-onset interval matters (i.e., meaning that it is within the temporal window of integration), we expect within-pair pitch relations to be easily extracted. This outcome would highlight the role of sounds’ offset for initiating the temporal window of integration. Accordingly, pitch information from the terminal part of the first tone of a pair were able to be linked with the pitch information of the second tone. Based on this assumption, we expect MMN amplitudes of comparable size in *Long Tone* and *Short*.

Additionally, the frontocentral MMN (Tervaniemi et al., 1994; Bendixen and Schröger, 2008) and the absent mastoidal MMN (Bendixen and Schröger, 2008) for abstract pitch relations whose extraction required the relation over temporal distances exceeding the integration period imply the engagement of different mechanisms in dependence of the timing. We used multi-channel electroencephalography (EEG)-recording and state-of-the-art variable resolution electromagnetic tomography (VARETA; Bosch-Bayard et al., 2001) to elucidate potential MMN differences across conditions.

One major advantage of measuring MMN in the current approach to test the hypotheses outlined before is that the influence of attention on the processing of abstract features can strongly be reduced as participants have no task related to the sounds. This enables studying automatic sound processing. In order to test whether the auditory system is in principle capable to detect the abstract rule violation, additional behavioral blocks (without ERP collection) were administered. Here, participants were asked to attend the auditory stimulation and actively detect the tone pairs with inverse pitch relation.

## MATERIALS AND METHODS

### PARTICIPANTS

Eighteen healthy volunteers (eight males) with self-reported normal hearing, aged 19–31 years (mean age: 23.1 years, SD: 2.9 years), participated in the experiment either for course credit or payment. Two additional volunteers had to be excluded from data analysis as they participated only in the first but not in the second experimental session. All subjects gave written informed consent according to the Declaration of Helsinki prior to the beginning of the measurements. We followed the ethical guidelines of the German Association of Psychology (“Deutsche Gesellschaft für Psychologie”).

### STIMULI AND PROCEDURE

Stimuli, generated via MATLAB^1^, were pairs of sinusoidal tones. We implemented three conditions. As a function of the corresponding condition the duration of the components of a pair, and thus of the pair itself, varied. In *Short* condition, two 90-ms tones, divided by a 20-ms silent interval, formed a 200-ms tone pair. In *Long Gap* condition, two 90-ms tones, divided by a 420-ms silent interval, formed a 600-ms tone pair. In *Long Tone* condition, the first tone of a pair lasted 490-ms, the second tone 90-ms. The two tones were divided by a 20-ms silent interval, resulting in a 600-ms tone pair. In each condition, tones included 10-ms rise and 10-ms fall times.

In different experimental blocks, the stimulation comprised either a rising or a falling rule formed by frequently (*p* = 87.5%) presented standard tone pairs with either increasing frequency relation or a decreasing frequency relation, respectively. To establish an abstract rule, absolute frequency values varied from trial to trial. Therefore, the frequency of the first tone of a pair was chosen randomly from 10-Hz steps in the interval of 600–1200 Hz. The frequency of the second tone was 26% higher or lower. 12.5 % of the tone-pairs (deviants) violated the respective rule inherent to the current stimulation by comprising the reversed frequency relation (**Figure 1**). Stimulus intensity was about 65 dB sound pressure level.

During EEG recordings subjects were seated in an acoustically attenuated and electrically shielded booth. They were instructed to watch a silent, self-selected and subtitled video while ignoring the auditory stimulation. The stimulation was run via MATLAB using the Cogent2000 toolbox^2^. The inter-pair interval, i.e., the temporal distance between the onsets of successive pairs, was 1200 ms. With regard to the stimulus sequence there was the constraint that deviant trials were preceded by at least four standard trials. Each block consisted of 160 tone pairs. For each condition, 10 blocks were administered: five blocks for the rising and five blocks for the falling rule.

Stimulation was run in two separate sessions at different days. In the first experimental session, stimuli from *Short* and *Long Tone* conditions were presented. The corresponding four different block types (two conditions, two rules) were presented with random permutation five times resulting in 20 experimental blocks. Pure stimulation time was 64 min. In the second session, *Long Gap* condition was run. Pure stimulation time was 32 min. The block types for rising and falling rules were presented in alternate order. Whether the stimulation started with blocks containing the rising or falling rule was counterbalanced across subjects.

At the end of the second session, participants took part in an active experiment without any EEG/electrooculography (EOG) recordings. If not otherwise reported stimuli and design were kept identical to those used in the passive experiment. The behavioral experiment consisted of two experimental blocks per condition. For each condition, one of the two blocks consisted of a tone sequence with a rising rule, the other one consisted of a tone sequence with a falling rule. Participants were informed about the rule (rising, falling) and the condition (*Short*, *Long Gap, Long Tone*) before starting the corresponding block type. Their task was to attend the auditory stimulation while continuously gazing at a cross displayed on the screen. Whenever they detected a violation of the abstract rule, participants’ task was to press as fast as possible the left mouse button with their right index finger. Before the behavioral experiment started, participants were trained to perceive the violation of the abstract rule. Individual correct and false responses, and reaction times (RTs) were recorded. At the end of each block, participants received visual feedback on their performance (correct response rate, false alarm rate, RT).

### DATA RECORDING AND ANALYSES

#### EEG data

***EEG recording.*** Using a BIOSEMI Active-Two amplifier EEG activity was recorded with Ag/AgCl electrodes from 32 standard channel locations according to the extension of the 10–20 electrode system (Chatrian et al., 1985), from left and right mastoid sites (LM and RM) and the nose. Two electrodes specific to the BioSemi acquisition montage (Common Mode Sense and Driven Right Leg) served for online reference and ground purposes. Additionally, EOG was measured with one electrode placed above the nasion and two electrodes below the outer canthi of the eyes. EEG and EOG signals were sampled at 512 Hz.

***EEG preprocessing.*** EEG analysis was performed with EEGlab (Delorme and Makeig, 2004) and EEProbe (ANT). Offline, data were referenced to the activity recorded at the nose and filtered with a 0.5–100 Hz band-pass FIR filter (length: 1856 points, Windowed sinc FIR, Kaiser beta 5.65; Widmann and Schröger, 2012). Thereafter, an automatic eye-movement correction (Schlögl et al., 2007) was applied. This was followed by filtering the data with a 20-Hz lowpass FIR filter (1857 points, Windowed sinc FIR, Kaiser beta 5.65). In *Short* condition, trial epochs were defined by a 200-ms baseline before the onset of the deviance (that is, before the second tone of a pair; cf. Paavilainen et al., 2003), and a 400-ms post-deviance interval. Thus, epochs had a length of 600 ms. In *Long Tone* and *Long Gap* conditions, epochs were defined by the onset of the first tone of the pair and a 400-ms post-deviance interval including a 200-ms baseline before the onset of the deviance. Thus, epochs in the two long conditions lasted 910 ms. Epochs with amplitude changes exceeding 100 μV on all EEG channels were rejected from further analysis. Also, the first standard of each block and the first standard after each deviant were excluded from further analysis. ERPs from stimulus sequences with rising and falling rules were collapsed in averaging. ERPs were averaged separately for each trial type (Deviant, Standard) and condition (*Short*, *Long Gap*, *Long Tone*). Difference waves were calculated by subtracting the ERP elicited to the standard from the ERP elicited to the corresponding deviant for each condition, respectively.

***ERP analysis.*** Mismatch negativity was measured at frontocentral (FC1, Fz, FC2) and mastoid sites (LM, RM) from the individual difference waves as the mean amplitude in 30-ms windows (**Table 1**) in the corresponding grand-average difference wave. Windows were chosen as the time interval around the mean latency of the MMN peak at the corresponding electrode sites. For statistical analysis mean amplitudes, measured at frontocentral electrode sites (FC1, Fz, FC2) and mastoids (LM, RM), respectively, were averaged. Presence of MMN was analyzed by applying one-sample, one-tailed Student’s *t*-tests to verify whether the mean amplitudes were significantly different from 0. One-way repeated measures analysis of variance (ANOVA) with the factor Condition was applied to test for differences in MMN amplitudes across condition. This was done separately for frontocentral and mastoidal sites.

**Table 1:** Mean MMN amplitudes measured at frontocentral (FC1, Fz, FC2) or mastoid sites (LM, RM), respectively, and the corresponding statistical results for each condition.

Condition	Electrode sites	30-ms window° centered on the peak latency (ms)	Amplitude in μV (SD)	*T* (df = 13)
*Short*	Frontocentral	132 ± 15	-1.08 (0.63)	-7.27***
	Mastoids	135 ± 15	0.67 (0.40)	7.20***
*Long Gap*	Frontocentral	116 ± 15	-0.98 (0.92)	-4.54***
	Mastoids	145 ± 15	0.24 (0.81)	1.27 n.s.
*Long Tone*	Frontocentral	116 ± 15	-0.73 (1.03)	-2.99**
	Mastoids	128 ± 15	0.65 (0.71)	3.93***

For assessing the MMN responses, mean amplitudes computed for deviant and standard ERPs were subtracted. To verify the presence of MMN, one sample one-tailed Student’s *t*-tests were applied to test against zero level (***p < 0.001, **p < 0.01; n.s. not significant). ^°^Note, that the window in which mean MMN amplitudes were measured are related to the onset of the deviance (i.e., the onset of the second tone of a pair starting at 110 or 510 ms following pair onset in *Short* or *Long Gap* and *Long Tone*, respectively).

***Topographic analysis.*** To evaluate differences in topographic distribution between MMN in *Short* and *Long Tone* or *Long Gap*, respectively, topographic maps of absolute voltage as well as scalp current density (SCD; see Giard et al., 2013 for a recent overview article) maps, were calculated for the three deviant-minus-standard difference waves. SCDs were obtained by utilizing a spherical spline interpolation of the scalp potential data with a maximum degree of the Legendre polynomials of 50 and order of splines of 4 (using the algorithm developed by Perrin et al., 1989). A smoothing parameter of 0.00001 was applied for estimating the SCDs.

***Tomographic analysis.*** To estimate cortical generators of MMN, a VARETA (Bosch-Bayard et al., 2001) was applied by utilizing the MATLAB toolbox Toolsbet and the BET Viewer (Neuronic). The mean of the amplitudes within the specified MMN time window (see **Table 1** for frontocentral electrodes) of the deviant ERP and the standard ERP, respectively, entered the analysis separately for each participant and condition. One subject was excluded from the tomographic analysis due to the inhomogeneous regularization parameter lambda for this subject (lambda controls the smoothness of the inverse solution and is automatically calculated for each of the participants). Note that the exclusion of this subject did not affect the main outcome of the VARETA analysis.

VARETA yields the spatial intracranial distribution of primary current densities (PCDs) in source space being best compatible with the amplitude distribution in electrode space. As possible sources of the MMN signal 3244 grid points (“voxels”) of a 3D grid (7 mm grid spacing) were used. This grid and the arrangement of 34 electrodes according to the international 10–20 system were placed in registration with the probabilistic brain atlas (developed at the Montreal Neurological Institute; Evans et al., 1993). This atlas provides an average of magnetic resonance imaging scans from 305 brains (“average brain”). Providing the information on where gray matter is expected in a mature young adult, the probabilistic brain atlas allows reducing artificial “ghost sources” by restricting the PCDs to the gray matter.

The MMN-PCD was determined by subtracting the PCD of the standard ERP from the PCD of the deviant ERP. To localize the MMN sources, statistical parametric maps of the MMN-PCD were constructed by utilizing a voxel-by-voxel Hotelling T^2^-test against 0 separately for each condition. To compare the MMN sources between the conditions *Short* and *Long Tone* or Short and *Long Gap*, respectively, the corresponding PCDs were contrasted by utilizing a voxel-by-voxel Hotelling T^2^-test. For all statistical parametric maps, a significance threshold of α = 0.0001 was utilized. Random field theory (Worsley et al., 1996) was applied to correct activation thresholds for spatial dependencies between voxels.

#### Behavioral data

Reaction times were measured relative to deviance’s onset (that is the onset of the second tone within a pair) exclusively for the correct responses. Early (<100 ms) or late (>1000 ms) responses were rejected from further analyses. Individual sensitivity indices (*d*′ values) were calculated, separately for each condition. In order to avoid infinite *d*′ values, 0.5 was added to the number of hits (responses to rule-violating tones/deviants) and to the number of false alarms (responses to rule-establishing tones/standards), and 1 was added to both the number of deviants and to the number of standards, before calculating hit- and false alarm rates (Macmillan and Creelman, 1991). By using this adjustment the highest sensitivity a participant could reach by responding 100% correct and 0% false would be 5.17. Behavioral data were analyzed with respect to *d*′ values and RTs.

## RESULTS

### EVENT-RELATED POTENTIALS

**FIGURE 2:**
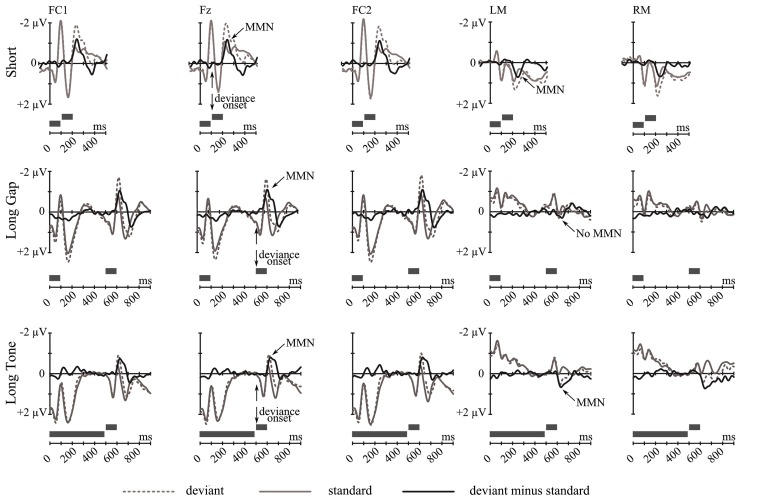
**Grand-average ERP waves at FC1, Fz, FC2, LM, and RM (from left to right) elicited by deviant tone-pairs (gray dotted line) and by physically identical standard tone-pairs (gray solid line), separately for each condition: *Short* (top), *Long Gap* (middle), and *Long Tone* (bottom).** The corresponding deviant-minus-standard difference wave (black) is depicted. Gray bars at the bottom of each diagram indicate an exemplary deviant tone pair. Vertical arrows point to the deviance onset. The elicitation of MMN is indicated. In each condition deviance onset is preceded by a 200-ms baseline interval (not highlighted).

**Figure 2** summarizes the grand-average ERP responses elicited by tone-pairs with an irregular pitch relation (deviants) and by the physical identical tone-pairs with regular pitch relation (standards) together with the deviant-minus-standard difference wave for each condition (*Short*, *Long Gap*, *Long Tone*) at frontocentral and mastoidal sites.

**FIGURE 3:**
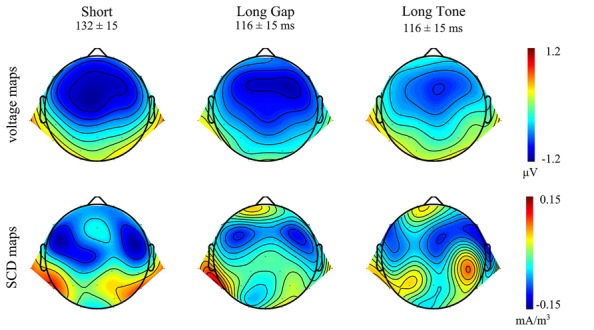
**Topographical distributions of the group-averaged MMN waveforms for each condition in the respective time windows.** *Top*: Voltage maps. *Bottom*: Scalp current density (SCD) maps.

In each condition, at frontocentral leads the MMN elicited in a given deviant trial reveals a larger negative deflection than in the corresponding standard trial. These negative deflections peak within the typical latency range of MMN, i.e., 110–140 ms after deviance onset, and show a frontocentral distribution that is typical for MMN (**Figure 3**). In *Short* and *Long Tone* condition, they were accompanied by a distinct polarity inversion at mastoid sites, which was less pronounced in *Long Gap* condition. For each condition, the presence of MMN at frontocentral sites and for *Short* and *Long Tone* at mastoid sites was statistically supported by the results of a one-sample two-tailed Student’s *t*-test that tested the mean amplitudes against 0. **Table 1** lists the MMN amplitudes and the corresponding statistical results.

The differences in MMN amplitudes were supported by the results of a one-way repeated measures ANOVA revealing a significant effect for Condition only at mastoid sites [mastoid sites: *F*(2,34) = 3.94, *p* < 0.03; frontocentral sites: *F*(2,34) = 0.97, *p* = 0.39]. Planned contrasts revealed that MMN amplitudes at mastoid sites were higher (more positive) for *Short* compared to *Long Gap* [*t*(17) = 2.56, *p* = 0.020] while MMN amplitudes for *Short* and *Long Tone* were of comparable size [*t*(17) = 0.1, *p* = 0.93].

Taken together, the ERP results and the voltage maps suggest that MMNs of comparable amplitudes were elicited at frontocentral sites in each condition, whereas MMN at mastoid sites were more pronounced in the conditions *Short* and *Long Tone* than in *Long Gap*.

The SCD maps (**Figure 3**, bottom) indicate MMN sources for all conditions in temporal areas; being visible for *Short* and *Long Tone* in both hemispheres while for *Long Gap* a respective source-sink pattern is mainly observable in the left hemisphere. The SCD maps additionally point at frontal MMN sources for *Long Gap* and *Long Tone*.

**FIGURE 4:**
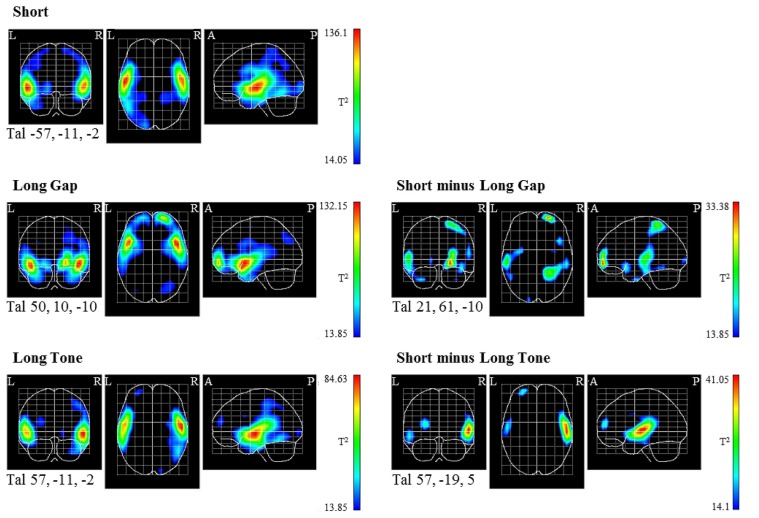
**Statistical parametrical maps of the tomographic analysis constructed on the basis of the “average brain” are shown for the coronal, axial, and sagittal plane (from left to right).** Note that all the slices of each plane are projected together onto one slice. The projection shows at each point of the slice the highest statistical T^2^ value of all slices in that plane. The view is similar as if the active areas were visible in a “glass head.” *Left column*: surface potential maps (SPMs) of the source reconstructions that were constructed by utilizing a voxel-by-voxel Hotelling T^2^-test against 0 separately for each condition. *Right column*: Source contrasts between conditions based on a voxel-by-voxel Hotelling T^2^-test. Tal, Talairach coordinates of the location in which the respective contrast is statistically strongest; R, right; L, left; A, anterior; P, posterior.

The statistical parametric maps (**Figure 4**), obtained via the VARETA approach, indicate PCD in the MMN time window that differ significantly from 0 in mainly the temporal areas of both hemispheres for all conditions (**Figure 4**, left). For each condition the strongest significant difference is located in supratemporal areas, though it varies across conditions with respect to the hemisphere as well as the position on the axial and/or coronal plane (Talairach coordinates of maximum peak for *Short*: *X*: -57, *Y*: -11, *Z*: -2; *Long Gap*: *X*: 50, *Y*: 10, *Z*: -10; *Long Tone*: *X*: 57, *Y*: -11, *Z*: -2). These data point to supratemporal MMN sources in the respective cortical region. The supratemporal sources are located more anterior in *Long Gap* compared to the sources in *Short* and *Long Tone* (**Figure 4**, left). In all conditions the supratemporal sources extend into the inferior frontal gyri as well as in parietal and occipital regions. Moreover, in *Long Gap* and *Long Tone* the PCDs differ significantly from 0 in frontal areas, pointing to frontal MMN sources in these conditions. In *Long Gap* the frontal sources are mainly located in the right lateral front-orbital gyrus. In *Long Tone* they are mainly located in the left middle frontal gyrus.

The direct comparison of the tomographic distributions of *Short* and *Long Gap* (**Figure 4**, right) shows significant differences in several areas such as temporal and frontal areas with the statistically strongest difference in the right lateral front-orbital gyrus (maximum peak: *X*: 21, *Y*: 61, *Z*: -10). An analog contrast between the tomographic distributions of *Short* and *Long Tone* also shows significant differences in several areas such as in temporal and frontal areas (i.e., middle frontal gyrus, left hemisphere), with the statistically strongest difference in the right supratemporal gyrus (right: *X*: 57, *Y*: -19, *Z*: 5).

Taken together, the topographic and tomographic distributions indicate MMN sources for all conditions mainly in supratemporal areas. They show a different spatial location in *Long Gap* compared to *Long Tone* and *Short*. In *Long Tone* and *Long Gap*, additional frontal areas are activated.

### BEHAVIORAL RESULTS

Behavioral data (*d*′ values, correct responses, false alarm rates, and RTs) obtained in response to the abstract rule violation are summarized in **Table 2**. In general, deviants were detected with moderate accuracy in all conditions.

**Table 2:** *d*′ Values, adjusted hit- and false alarm rate, and reaction times to rule-violating pitch relations obtained in the active deviant detection task for each condition.

Condition	*d*′ Value (SD)	Hits in % (SD)	False alarms in % (SD)	Reaction times in ms (SD)
*Short*	2.77 (1.29)	72.90 (19.78)	5.24 (6.74)	455 (60)
*Long Gap*	3.22 (1.39)	73.58 (24.10)	2.17 (3.04)	404 (57)
*Long Tone*	3.02 (1.32)	75.47 (19.71)	4.01 (6.23)	418 (55)

*Standard deviations (SD) are given in parentheses.*

The results of a two-way repeated measures ANOVA that tested for differences in *d*′ values and RTs, respectively, across conditions showed significant main effects [*d*′ values: *F*(2,34) = 5.22, *p* = 0.011; RTs: *F*(2,34) = 13.58, *p* < 0.001]. Planned contrasts of *d*′ values revealed that participants responded more sensitive to deviant tone-pairs in the *Long Gap* than in the *Short* condition [*t*(17) = 3.11, *p* = 0.006]. No other contrast revealed significant differences in *d*′ values. Moreover, planned contrasts of RTs revealed that participants responded faster to deviants in the conditions *Long Gap* and *Long Tone* compared to the *Short* condition [*t*(17) = -5.37, *p* < 0.001 and *t*(17) = -3.95, *p* = 0.001, respectively].

## DISCUSSION

The current study tested whether temporal aspects inherent to the auditory input play a crucial role for the central auditory system to automatically extract invariant pitch relations from a varying auditory context. In the following, we will first shed light on the outcome of the behavioral approach, in which sounds were task-relevant. This is to confirm that rule violating events were in principle available for the deviance detection system. Thereafter, we discuss the data set obtained with the passive listening approach utilizing the ERP method to focus on the automatic processing of task-irrelevant abstract pitch relations.

### THE IMPACT OF TIMING ON THE PROCESSING OF TASK-RELEVANT PITCH RELATIONS

As can be inferred from the behavioral data obtained for each condition (*Short*, *Long Gap*, *Long Tone*) the auditory system can in principle extract task-relevant pitch relations. This has already been shown in previous reports (e.g., van Zuijen et al., 2006; Schröger et al., 2007; Bendixen and Schröger, 2008). For instance, the hit rate in condition *Short* (about 73%, **Table 2**) resembles the one obtained with a similar approach conducted earlier (72%, Schröger et al., 2007).

Importantly, the impact of timing on the extraction of task-relevant abstract pitch relations has not been systematically studied up to now. In this respect, the data (sensitivity indices and RTs) obtained in the conditions *Long Gap* and *Long Tone* extend results from previous reports (e.g., van Zuijen et al., 2006; Schröger et al., 2007; Bendixen and Schröger, 2008). The current data reveal differences in sensitivity of deviance detection and its RT: for short onset-to-onset intervals (*Short* condition) RTs are prolonged (compared to *Long Gap* and *Long Tone*) and sensitivity is reduced (compared to *Long Gap*). As the pitch of the first tone within a pair could be used to predict the pitch of the second tone, the detection of task-relevant pitch relations seems to benefit from a longer preparation time (in *Long Gap* and *Long Tone*).

Importantly, long onset-to-onset within-pair intervals did not lead to an impaired extraction of task-relevant abstract features. The latter outcome extends comparable findings obtained for the extraction of task relevant simple features (Weise et al., 2010, 2012; Timm et al., 2011; Hoonhorst et al., 2012) in which the temporal constraint also applied only under passive, but not under active listening condition.

### THE TEMPORAL CONSTRAINT DOES NOT IMPAIR THE AUTOMATIC ENCODING OF TASK-IRRELEVANT ABSTRACT PITCH RELATIONS

As expected, irregular abstract pitch relations elicited a distinct MMN when the respective tones were separated by a short onset-to-onset within-pair interval (*Short*). Comparable findings obtained with a similar approach have already been reported before (Saarinen et al., 1992; Paavilainen et al., 2003; Carral et al., 2005; van Zuijen et al., 2006; Schröger et al., 2007). Interestingly and in contrast to the temporal-constraint hypothesis (Atienza et al., 2003; Näätänen et al., 2004; Grimm and Schröger, 2005; Sussman and Gumenyuk, 2005; Hoonhorst et al., 2012), we found an undiminished MMN amplitude at frontocentral sites even for long onset-to-onset within-pair intervals (*Long Gap*, *Long Tone*). This pattern of results supports previous data (Tervaniemi et al., 1994; Bendixen and Schröger, 2008) showing that abstract pitch relations between adjacent tones can be automatically extracted when the onset-to-onset interval exceeds the temporal window of integration. Crucially, the current data set extends earlier findings (Tervaniemi et al., 1994; Paavilainen et al., 2003; Bendixen and Schröger, 2008) by showing comparable frontocentral MMN amplitudes irrespective of the systematic variation in timing (*Short*, *Long Gap*, *Long Tone*; see next section for a deeper discussion). It remains to be tested, whether abstract pitch relations can be automatically processed even for longer intervals spanning up to several seconds. Behavioral data from a different approach, which also tapped into automatic processing, supports this notion (Demany and Ramos, 2005).

### DIFFERENT MECHANISMS ENGAGED IN THE AUTOMATIC PROCESSING OF ABSTRACT PITCH RELATIONS

Unlike the amplitudes of the frontocentral MMN, those of the mastoidal MMN differed across conditions. This suggests that in dependence of the timing identical pitch relations are encoded by different neuronal assemblies. More specifically, abstract pitch relations with short silent within-pair intervals (*Short* and *Long Tone*) elicited a distinct mastoidal MMN. Crucially, when the silent within-pair interval exceeded the limits of the temporal window of integration (*Long Gap*), the mastoidal MMN was (statistically) eliminated (cf. **Figure 2**; **Table 1**). This cannot simply be explained due to a low signal-to-noise ratio (Paavilainen, 2013). A similar ERP-outcome to that in *Long Gap* was obtained in a dynamic approach in which auditory abstract rules (i.e., pitch relations) constantly emerged and vanished. Violations of those rules elicited a frontocentral MMN but no mastoidal MMN though the onsets of successive tones were separated by 1400 ms (Bendixen and Schröger, 2008).

The current tomographic analysis may explain the absent mastoidal MMN (*Long Gap*; Bendixen and Schröger, 2008). It shows supratemporal activation not only for pairs with a short silent interval (*Short* and *Long Tone*) but also for pairs with a long silent interval (*Long Gap*). Crucially, in *Long Gap*, the supratemporal activation pattern is located more anterior than that in *Short* and *Long Tone.* That is, due to its different source distribution one might infer that the supratemporal generators in *Long Gap* have a different orientation (cf. Alho, 1995; Giard et al., 1995) which is why their activity is not visible at the mastoidal sites in the ERP data. This explanation seems more reasonable than assuming that solely frontal generators are engaged in the processing of pitch relation when events are separated by long silent intervals (*Long Gap*; Bendixen and Schröger, 2008). The current findings are also in line with the notion that the frontocentral MMN receives contribution from both frontal and supratemporal generators (Alho, 1995; Deouell, 2007).

Besides differences in the distribution of supratemporal activation across conditions the outcome of the tomographic analysis revealed also variations in frontal activation. In particular, when the within-pair SOA exceeded the temporal integration period (*Long Gap*, *Long Tone*) frontal activation was observed. In contrast, when the SOA was short (*Short*), only supratemporal activation was observed. The diverging topographic MMN distributions across conditions contribute to the evidence for several distinct generator structures of the deviance detection system (Opitz et al., 2002; Doeller et al., 2003; for a reviews, see Deouell, 2007). Concerning the functional interpretation, the current findings support previous reports claiming that rule violations can be detected at several different anatomical levels. Recent reports show, for instance, that simple rule violations are already processed at subcortical levels (Grimm and Escera, 2012; Escera et al., 2013), whereas the violation of more complex features (e.g., feature conjunctions) require cortical structures to be detected (Althen et al., 2013). Thus, in first place, deviance detection is undertaken by neuronal structures at the lowest possible anatomical level in the auditory pathway and, if required, also by neuronal assemblies of a hierarchically higher structure. Accordingly, in the current study posterior regions of the supratemporal cortex are engaged when the silent within-pair interval is short (*Short*, *Long Tone*). This supports previous findings indicating MMN generators at the auditory cortices which process abstract features (Schröger et al., 2007; for an overview article, see Paavilainen, 2013). If the silent within-pair interval exceeds the integration period (*Long Gap*), higher auditory areas (here: anterior parts of the supratemporal gyrus) might be required to extract identical abstract pitch relations. Additional frontal generators are involved in the processing of abstract pitch violations when timing exceeds the temporal window of integration (*Long Gap*, *Long Tone*). Noteworthy, we found the frontal activation to be even stronger when not only the stimulus-onset-asynchrony (*Long Tone*) but also the silent within-pair interval exceeds the critical integration period (*Long Gap*). The involvement of frontal generators is compatible with data obtained via electrophysiological recording and anatomical tracing in anesthetized rhesus macaques indicating auditory-frontal pathways (Romanski et al., 1999). Further, it matches the view that the detection of abstract rule violations may rely on a neuronal network with responsive neurons in the auditory fields of the temporal lobes as well as in the frontal lobe (Korzyukov et al., 2003).

To the best of the authors’ knowledge this is the first data set highlighting the stronger engagement of the more anterior supratemporal areas as well as the frontal cortex in abstract pitch processing when the SOA and/or the silent interval within a pair is increased. Though a previous study also failed in obtaining a mastoidal MMN for abstract features when the SOA between adjacent events was beyond 1 s (e.g., Bendixen and Schröger, 2008) this report does not provide tomographic analysis focusing on potential frontal MMN sources. The current pattern of results asks for further research on the specific functional role of the frontal cortex in auditory deviance detection (cf. Deouell, 2007) by combining ingenious designs with methods allowing source analysis.

### THE ROLE OF SOUND OFFSET IN ABSTRACT PITCH PROCESSING

The differences in supratemporal generator location in *Long Gap* vs. *Long Tone* highlight another aspect of the extraction and representation of auditory information. The diverging results might stem from the fact that in *Long Tone* more auditory information is available that can be used by the sensory supratemporal generators to integrate adjacent events. Studies on the temporal constraint in the representation of pure tones (Näätänen et al., 2004; Grimm and Schröger, 2005; Hoonhorst et al., 2012) revealed that later parts of a sound (>350 ms) are not (or less) accessible for the comparison-based deviance detection system. However, given that auditory transients can initiate integration anew, the undiminished mastoidal MMN with its sources in the posterior parts of the supratemporal cortex in *Long Tone* to a late violation (with respect to pair onset) suggests that not only sound-/pair-onset (Yabe et al., 1997; Näätänen and Winkler, 1999; Atienza et al., 2003) or a pitch transition within tones (Weise et al., 2012) can trigger integration, but also sound offset. Note that this interpretation also assumes that the more anterior parts of supratemporal cortex, activated in *Long Gap*, do reflect a higher-order (e.g., categorical) integration mechanism, whereas activations in more posterior supratemporal regions might represent a more sensory based integration mechanism. The latter seems to be triggered by sound onset, and similarly by sound offset.

The role of sound offset in this context has not been reported before. This is because previous approaches focusing on the impact of timing on auditory processing varied the offset-to-onset interval together with the onset-to-onset interval between adjacent tones (e.g., Alain et al., 1994; Atienza et al., 2003; Paavilainen et al., 2003; Sussman and Gumenyuk, 2005). This did not allow disentangling the distinct impact of sound onset and sound offset. The functional role of sound offset received in general less attention to date. This is probably because of the fact that sounds onset is processed by a larger population of neuronal elements than its offset (Phillips et al., 2002; Middlebrooks, 2005; Wang et al., 2005). This is reflected, for instance, in larger ERPs to the onset than to the offset of a sound (Yamashiro et al., 2009, 2011). However, as can be inferred from the current outcome this does not necessarily mean that the offset of a sound is in general less relevant for auditory processing. This notion receives support from the fact that offset timing is at least as accurately processed as that of sound onset (reflected in comparable or even shorter latencies for sound offset (Hari et al., 1987; Pantev et al., 1996; Yamashiro et al., 2009, 2011). Anyway, to strengthen the current link between sound offset and sensory integration and to shed more light on the functional role of sound offset in auditory scene analysis in general, further research is required.

Note, from the findings for long tone pairs with short silent intervals (*Long Tone*), it cannot be generalized that the offset of the first tone (or the offset-to-onset timing, respectively) has a larger impact on the extraction of abstract pitch relations than the onset of the first tone (or the onset-to-onset timing). For instance, when considering short tone pairs (*Short*) the offset (or the offset-to-onset timing) might play a minor role as the complete information of this tone pair falls within the temporal window of integration initiated by the onset of the first tone, enabling the extraction of the pitch relation. This is in line with findings showing that the offset-related neural response diminishes as the tone’s duration decreases (Näätänen and Picton, 1987; Yamashiro et al., 2011).

Given that the silent within-pair interval and the silent between-pair interval were more similar in *Long Gap* (420 vs. 600 ms, respectively) than in *Short* (20 vs. 1000 ms) and *Long Tone* (20 vs. 600 ms) one might argue that this weakens the interpretation concerning the functional role of sound offset. More precisely, the different MMN source localizations in *Long Gap* vs. *Short* and *Long Tone* might rather be explained by an increased processing demand when the corresponding temporal intervals within the stimulation are more similar to each other (*Long Gap*) making the pitch relation more difficult to extract. Indeed, there is behavioral evidence that not only the absolute time matters for the decay of memory for pitch, but that the temporal distinctiveness (i.e., the inter-pair interval relative to the inter-tone interval) has a genuine impact on pitch memory (Cowan et al., 1997). However, the current behavioral data rather argue against this interpretation in showing that pitch extraction was not diminished (at least when sounds are task-relevant). Nevertheless, though the sensitivity of MMN elicitation has often been linked to the perceptual discrimination ability (Tiitinen et al., 1994; for a review, see Näätänen and Winkler, 1999) this does not necessarily hold in every case. It has, for instance, been shown that certain rule violations can be extracted when task-relevant whereas when task-irrelevant they cannot (indicated by the absent MMN; Hoonhorst et al., 2012). The other way around is also possible: rule-violations elicited MMN when task-irrelevant while the perceptual discrimination sensitivity was poor when violations were task-relevant (van Zuijen et al., 2006; Paavilainen et al., 2007). Thus, the functional role of sound offset as well as the impact of the similarity of the temporal intervals within a tone-pair paradigm (silent within-pair interval and the silent between-pair interval) on the extraction of pitch relations seems to need more research, especially when sounds are non-attended and task-irrelevant.

### TIMING DIFFERENTIALLY IMPACTS THE PROCESSING OF COMPLEX AUDITORY EVENTS

Interestingly, despite the fact that timing impacts the detection of rule-violating abstract pitch relations, abstract regularities were in principle extractable under all timing conditions. This is in contrast to recent reports (Atienza et al., 2003; Sussman and Gumenyuk, 2005) in which timing had an impact on rule extraction and thus deviance detection. For instance, the differing pitch of the fifth tone in a repeatedly presented five-tone pattern (A-A-A-A-B) did only elicit MMN when the timing between the successive events was beyond the limits of the temporal window of integration as here the A tones were extracted as invariant events to form the rule while the B tone was the deviating event. On the other hand, when timing was kept within the integration range, the complete five-tone pattern served as invariant event. In that case, no rule violating event was present (Sussman and Gumenyuk, 2005). From the diverging MMN outcome of different approaches (pattern-approach: Atienza et al., 2003; Sussman and Gumenyuk, 2005 vs. current approach) it becomes clear that – depending on the stimulation parameters – timing differentially impacts how complex auditory events are automatically processed. If the timing between adjacent events is outside the integration period, this does not necessarily lead to an impaired extraction of auditory rules.

## CONCLUSION

The current results confirmed that abstract within-pair pitch relations can automatically be extracted even when the stimulus-onset-asynchrony between the to-be-linked events exceeds the temporal window of integration. The present study revealed that mainly more posteriorly located parts of the supratemporal cortex are activated when the silent within-pair interval does not exceed the integration period. If the silent within-pair interval does exceed the integration window more anterior parts of the supratemporal cortex as well as frontal generators come into play. Thus, depending on the timing, different mechanisms are engaged to encode the pitch relations. We suggest that the one reflected in more anterior activity is more categorical, while the one reflected in more posterior activity is more sensorial in nature. The data, further, emphasize the role of sounds’ offset for auditory processing in that not only sound onset but also sound offset is a potential candidate triggering an integration process.

## AUTHOR CONTRIBUTIONS

Annekathrin Weise, Sabine Grimm, and Erich Schröger conceived and designed the experiment. Annekathrin Weise performed the experiment, analyzed the data, and drafted the work. Nelson J. Trujillo-Barreto and Sabine Grimm guided the VARETA analyses. Annekathrin Weise, Sabine Grimm, Erich Schröger, and Nelson J. Trujillo-Barreto revised the work critically and approved the final version of the manuscript before its submission. Annekathrin Weise, Sabine Grimm, Erich Schröger, and Nelson J. Trujillo-Barreto agree to be accountable for all aspects of the work in ensuring that questions related to the accuracy or integrity of any part of the work are appropriately investigated and resolved.

## Conflict of Interest Statement

The authors declare that the research was conducted in the absence of any commercial or financial relationships that could be construed as a potential conflict of interest.

## Footnotes

1 http://www.mathworks.com

2 http://www.vislab.ucl.ac.uk/cogent_2000.php
